# Design of Intelligent Neuro-Supervised Networks for Brain Electrical Activity Rhythms of Parkinson’s Disease Model

**DOI:** 10.3390/biomimetics8030322

**Published:** 2023-07-21

**Authors:** Roshana Mukhtar, Chuan-Yu Chang, Muhammad Asif Zahoor Raja, Naveed Ishtiaq Chaudhary

**Affiliations:** 1 Department of Computer Science and Information Engineering, Graduate School of Engineering, Science and Technology, National Yunlin University of Science and Technology, Yunlin 64002, Taiwan; d11110209@yuntech.edu.tw; 2 Department of Computer Science and Information Engineering, National Yunlin University of Science and Technology, Yunlin 64002, Taiwan; chuanyu@yuntech.edu.tw; 3 Future Technology Research Center, National Yunlin University of Science and Technology, Yunlin 64002, Taiwan; rajamaz@yuntech.edu.tw

**Keywords:** Parkinson’s disease, neuro-supervised networks, intelligent computing, Bayesian regularization, Levenberg–Marquardt

## Abstract

The objective of this paper is to present a novel design of intelligent neuro-supervised networks (INSNs) in order to study the dynamics of a mathematical model for Parkinson’s disease illness (PDI), governed with three differential classes to represent the rhythms of brain electrical activity measurements at different locations in the cerebral cortex. The proposed INSNs are constructed by exploiting the knacks of multilayer structure neural networks back-propagated with the Levenberg–Marquardt (LM) and Bayesian regularization (BR) optimization approaches. The reference data for the grids of input and the target samples of INSNs were formulated with a reliable numerical solver via the Adams method for sundry scenarios of PDI models by way of variation of sensor locations in order to measure the impact of the rhythms of brain electrical activity. The designed INSNs for both backpropagation procedures were implemented on created datasets segmented arbitrarily into training, testing, and validation samples by optimization of mean squared error based fitness function. Comparison of outcomes on the basis of exhaustive simulations of proposed INSNs via both LM and BR methodologies was conducted with reference solutions of PDI models by means of learning curves on MSE, adaptive control parameters of algorithms, absolute error, histogram error plots, and regression index. The outcomes endorse the efficacy of both INSNs solvers for different scenarios in PDI models, but the accuracy of the BR-based method is relatively superior, albeit at the cost of slightly more computations.

## 1. Introduction

**Figure 1:**
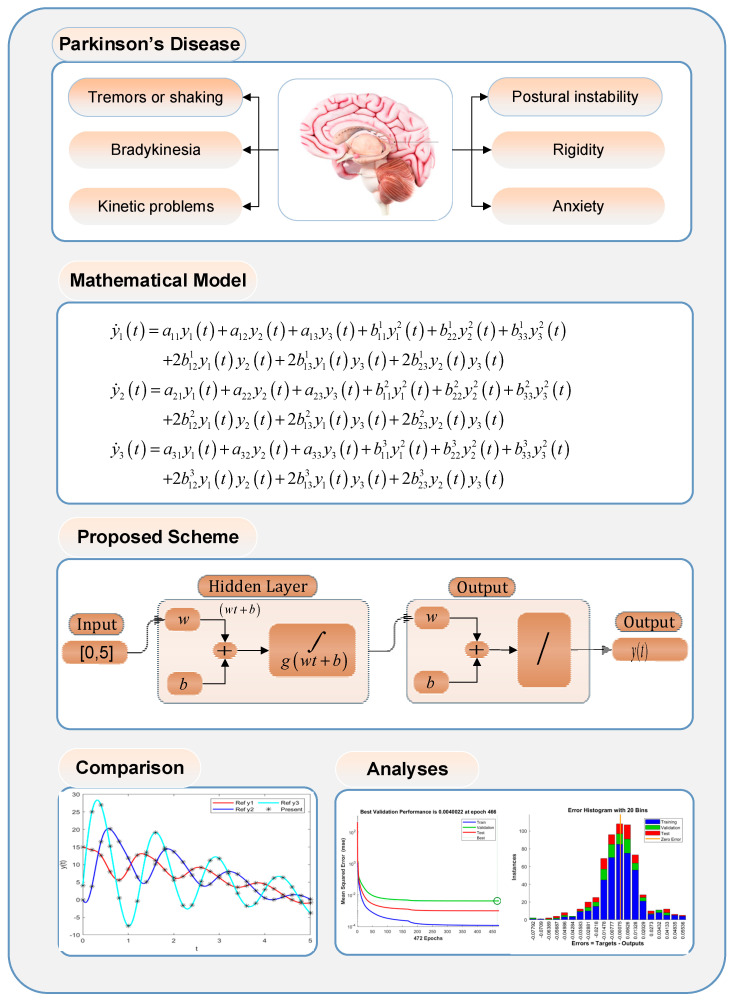
Graphical abstract of the PDI study using INSNs.

Parkinson’s disease illness (PDI) is a neurological disorder normally caused by an early significant death of dopaminergic neurons, and the resulting deficiency of dopamine within the basal ganglia results in movement disorders [1]. PDI patients may suffer from tremors/shaking, kinetic problems, postural instability, and rigidity and anxiety, as highlighted in Part 1 of the graphical abstract provided in Figure 1. In 2016, around 6.1 million people were affected by the PDI [2], and a rapid increase in PDI patients has been observed in the past two decades [3,4]. The current therapeutic treatments for PDI are based on restoring dopamine levels. These remedies are helpful in providing symptomatic relief to PDI patients, but they are not disease modifying, and, therefore, PDI has remained incurable [5]. Mathematical modeling of PDI may help in better understanding the dynamics of the disease and, thus, improved treatments for its recovery. Different mathematical models of PDI have been proposed [6]. For instance, in [7], Anninou et al. developed a mathematical model for PDI by exploiting the concept of fuzzy cognitive maps, and a generic algorithm was proposed based on nonlinear Hebbian learning techniques. Recently, a wide use of artificial intelligence methodologies for modeling different diseases has emerged [8,9,10], but, as per our exhaustive search, these methodologies are not yet exploited to study the dynamics of PDI. Thus, it seems promising to exploit the well-established strength of machine learning and artificial intelligence techniques to study the dynamics of PDI. 

The objectives of the current investigation are as follows:

- Study the dynamics of a mathematical model for PDI, governed with three differential classes to represent the rhythms of brain electrical activity that are measured at different locations of the cerebral cortex.
- Construct intelligent neuro-supervised networks (INSNs) by exploiting the knacks of multilayer structure neural networks backpropagated with the Levenberg–Marquardt (LM) and the Bayesian regularization (BR) approaches.
- Optimize the mean squared error based fitness function for sundry scenarios of PDI models by the variation of sensor locations to measure the impact of the rhythms of brain electrical activity.
- Compare the outcomes on the basis of exhaustive simulations of the proposed INSNs via both LM and BR methodologies with reference solutions of PDI models by means of learning curves on MSE, adaptive control parameters of algorithms, absolute error, histogram error plots, and regression index.

The structure of the remaining paper is as follows: Section 2 presents the details of the related works; Section 3 provides the mathematical model of PDI along with a description of the proposed INSNs; Section 4 discusses the simulation results for different scenarios of PDI; and Section 5 concludes the study by noting some potential future research directions.

## 2. Related Works

In PDI, the freezing of gait of patients is a common problem, referring to sudden/temporary inability to initiate or continue walking, and the PDI patient feels as if his or her feet are glued to ground when this occurs [11]. The freezing episodes often occur in PDI patients during the gait initiation or when turning, and they may considerably affect mobility/independence. Although the exact reason for freezing in PDI is not yet fully understood, it is widely believed that it results from a combination of motor/cognitive factors, such as (i) motor fluctuations, where the person experiences periods of good mobility—on periods and periods of poor mobility i.e., only off periods; (ii) dual-task interference, i.e., performing dual tasks, such as talking when walking or carrying an object, can enhance the risk of freezing; and (iii) emotional factors, such as anxiety and stress, can trigger or may worsen the freezing of gait. Parakkal et al. investigated the freezing of gait in PDI patients and suggested an ankle push off model [11], where a simplified neuromechanical model of gait is used for observing the variability and freezing in PDI. The mathematical model presented in [11], composed of the stance-leg, demonstrated an inverted pendulum (IP) operated by the ankle, and pushes off forces through the trailing-leg and pathological forces from the plantar flexors of the stance-leg. Further, the effect on walking of the swing-leg is modeled in a biped model (BM), while freezing and irregular walking is studied in both the BM and IP model. The plantar flexors (PF) correspond to the swing-leg pushing the center-of-mass forward, and the PF correspond to the stance-leg producing opposing torque. The study conducted in [8] demonstrated that the opposing forces produced by PF can persuade freezing, and it also explained the gait irregularities that are closer to freezing, such as step length reduction and irregular walking patterns.

Heyete et al. [12] presented the Bayesian mathematical model (BMM) to identify predictors of long-term motor/cognitive results and the progression rate in PDI. A BMM of motor/cognitive outcomes in PDI may aid understanding of the complex interactions among various factors and predict the progression of the disease. BMM exploits the concepts of probability and integrates data from multiple sources, including clinical assessments, demographic details, and neuroimaging results, to predict the different motor and cognitive outcomes. Belozyotov et al. [13] presented a mathematical model to study the behavior of PDI through the EEG signal of the patient taken at three different locations of the cerebral cortex (CC), considered as a three-time series describing the behavior of the disease curve in a three-dimensional phase-space, and then the 3D system of quadratic differential equations was constructed, whose solution provides the disease curve. Further, in [13], chaotic dynamics have been observed in the PDI mathematical model that can capture the complex interactions among the neurons and how their activity evolves over time. The chaotic attractors formed by the CC signals give information about the normal process or disease progression, depending on the nature of the chaotic graph.

Borah et al. [14] presented the fractional order model of PDI by exploiting the strong mathematical foundations of fractional calculus that allow real order differentiation or integration to be taken through generalizing the conventional integer order calculus. The design of appropriate controllers to control the chaos in the bio-mathematical models, including the PDI presented, enables stable performance to be attained. Further, the design of anti-controllers is also demonstrated in [14] for generating chaos when turbulence is required. Detailed analyses of the fractional order PDI model involving chaos are conducted in [14], where absence of chaos reflects the onset of the disease, and where anti-control schemes through linear state feedback, sliding mode, and single-state sinusoidal feedback are developed.

The intelligent computing approaches are introduced to effectively model or optimize different engineering, mathematical, and applied sciences problems [15,16,17,18]. In [19], a convolutional neural network (CNN) framework is introduced for emotion recognition that takes input from the extracts of mel scale spectrogram, chromagram, Tonnetz representation, mel frequency cepstral coefficients, and spectral contrast features through speech files. In [20], the estimated yield of soya bean crops under drought conditions is studied through imagery from an unmanned aerial vehicle and CNN. Further, it is demonstrated that the fusion of 1-D and 2-D inputs in a CNN-based deep learning model enhances the estimated accuracy. In [21], an improved denoising autoencoder (DAE) is developed that integrates the concept of confidence level in conventional DAE for fast and accurate recommendations in recommender systems, which are required in the E-commerce industry to provide reliable recommendations to the users. The DAE structure proposed in [21] is effectively applied to Movie Lens 100 K and 1 M datasets with outstanding performance compared to the standard DAE structures in terms of precision, recall, and MAP metrics. In [22], swarm intelligence is exploited for the identification of fractional order nonlinear autoregressive exogenous systems through established strength of particle swarm optimization (PSO). In [23], a hybrid bi-directional gated recurrent unit (BiGRU) and bi-directional long-term short-term memory (BiLSTM) are presented for electricity theft detection in smart grids with preprocessing through feature engineering. Before using the BiGRU and BiLSTM for classification, the data imbalance issue is solved using a K-means minority oversampling scheme such that the balanced data are given as an input to the BiGRU and BiLSTM models for better classification accuracy. In [24], the fractional calculus concepts are incorporated in the optimization mechanism of PSO to enhance its optimization strength for effective parameter estimation of nonlinear Hammerstein autoregressive exogenous systems. Further, the key term separation principle is introduced in the PSO to accurately estimate the actual parameters of the Hammerstein nonlinear system by avoiding the redundant parameters. [25] present marine predators based optimization heuristics for a parameter estimation of Hammerstein output error systems. The marine predator is a recently introduced swarm intelligence optimization approach that mimics the behavior of predators for catching prey through Brownian and Levy distributions for estimating the optimum communication between predator and prey. In [26], the weather classification model through the hybrid CNN and generative adversarial network is developed for photovoltaic power forecasting. In [27], knacks of feedforward artificial neural networks (FANN) optimized with the Levenberg–Marquardt algorithm are presented for analysis of the power law fluidic problem of moving wedge and flat plate model. In [28], FANN optimized with the Bayesian regularization algorithm are exploited for peristaltic motion of a third grade fluid in the planar channel. In [29], FANN optimized with the Levenberg–Marquardt algorithm are proposed to study the dynamics of multi-walled carbon nanotubes coated with gold nanoparticles with a different velocity slip in curved channel peristaltic motion. In [30], the efficacy of ANN optimized with the Levenberg–Marquardt and the Bayesian regularization algorithms is analyzed for the Cattaneo–Cristov heat flux model with biconvection nanofluid flow. In [31], intelligent algorithms and control schemes are presented for battery management in electric vehicles with details of current advancement, major challenges, and future prospects in the domain of battery management systems. In [32], a comprehensive survey of the applications of the intelligent transportation systems is presented in the context of big data, with identification of the research gaps and potential future research directions in the domain of intelligent transportation systems. In [33], a variety of issues related to interoperability in the Internet of Things (IoT) are discussed, such as searching/processing IoT, implementing, modeling event, and workflow processes. In [34,35], automatic detection of motor imagery EEG signals is obtained for robust brain computer interface systems. In [36], the recognition of alcoholic EEG signals is performed using CNN and the concept of geometrical features. The graphical features are one of the newest approaches for identifying underlying patterns of EEG signals, and they are used for effective depression detection [37] as well as seizure recognition [38].

There are a few intelligent computation algorithms. These include a chimp-inspired optimization scheme [39], i.e., an intelligent optimization algorithm effectively exploited to solve different problems with reasonably accuracy through providing a good balance in the exploration and exploitation phases; a Kohonen neural network [40], i.e., an unsupervised self-organizing (SO) competitive neural network that performs automatic clustering and that updates the weights of the network through SO feature mapping with effective application to intrusion detection of the network virus; and a Mayfly algorithm [41], i.e., a swarm intelligence-based heuristic approach, applied to successfully solve different engineering optimization problems, including the asymmetric traveling salesman problem, due to the features of population diversity and enhanced local search capability. Others include a simplified slime mould algorithm [42], i.e., a modified version of the slime mould heuristic, with an introduction of enhanced adaptive oscillation for better exploration capability during the early search phase, with application to wireless sensor network optimization problems; a code pathfinder algorithm [43], i.e., a discrete complex code pathfinder heuristic for an efficient solution to the optimization problem of wind farm layout through an improved exploration capability; and a firefighting strategy based marine predators approach [44], i.e., an improved variant of marine predator heuristic through an introduction of opposition-based learning for more uniform initial population and adaptive weight factor for creating balance between exploration/exploitation capabilities to effectively handle the forest fire rescue issues. More of these intelligent computer algorithms include a chaotic grey wolf optimizer [45], i.e., a modified grey wolf optimizer by incorporating the concepts of chaotic maps and adaptive convergence factor for robust and accurate parameter estimation of control autoregressive systems; a subtraction average based optimizer [46], i.e., an optimization approach inspired by the subtraction average of searchers agents for the position updates of the particles in the search space; and an enhanced dragonfly heuristic [47], i.e., enhanced version of dragonfly algorithm with an improved mechanism of global search and a local search for the four color map problem. There are also two others: a non-dominated sorting genetic algorithm [48], i.e., a modified variant of genetic algorithm with special congestion approach and adaptive crossover scheme to effectively solve multi objective and multi modal optimization problems, and, lastly, a green anaconda optimizer [49], i.e., an optimization heuristic that mimics the natural behavior of the green anacondas to solve various benchmark optimization challenges.

The intelligent computing-based methodologies have been proposed for bioinformatics and biotechnology applications as well. These include a combination of a graph neural network and CNN for efficient breast cancer classification [50]; deep learning and transfer learning through regional CNN for white blood cell detection [51]; and a fine-tuned neural network and long-term short-term memory-based neural network for skin disease [52]. They also include a convolutional autoencoder and transfer learning-based scheme for Alzheimer’s disease visualization [53]; a perceptron neural network for bacterial behavior programming [54]; and a deep neural architecture with generative adversarial network for brain tumor classification [55]. In addition, they include a deep neural network for epidemic prediction of COVID disease [56]; deep learning for sequential analysis of biomolecules [57]; elastic net and neural networks for the identification of plant genomics [58]; data mining and machine learning algorithms based on spectral clustering, random forest, and neural networks for cancer diagnosis through gene data [8]; and a stacking ensemble model based on an auto-regressive integrated moving average, exponential smoothing, a neural network autoregressive, a gradient-boosting regression tree, and extreme gradient boost models for infectious diseases [9]. Finally, there are supervised machine learning algorithms for lung disease detection, respiratory sound analyses, and so on [10]. Motivated by the widespread applications of the artificial intelligence methodologies, this study investigates exploiting the artificial intelligence techniques to study the dynamics of PDI.

## 3. Proposed Methodology

Before developing the INSNs, first the mathematical model of the PDI is introduced in this section. Let the rhythms $x_{1}\left(t_{i}\right),x_{2}\left(t_{i}\right),\ldots ,x_{k}\left(t_{i}\right),i=1,2,\ldots ,N$ of cerebral activity at *k* point of cerebral cortex be measured by EEG and defined as [13]:

(1) $$\begin{array}{c} x_{1}\left(t_{i}\right)=y_{1}\left(t_{i}\right)+\varepsilon_{1}\left(t_{i}\right),\\ x_{2}\left(t_{i}\right)=y_{2}\left(t_{i}\right)+\varepsilon_{2}\left(t_{i}\right),\\ \vdots \\ x_{k}\left(t_{i}\right)=y_{k}\left(t_{i}\right)+\varepsilon_{k}\left(t_{i}\right), \end{array}$$

where $y_{1}\left(t_{i}\right),y_{2}\left(t_{i}\right),\ldots ,y_{k}\left(t_{i}\right)$ shows the discrete approximation of $y\left(t\right)={\left[y_{1}\left(t\right),y_{2}\left(t\right),\ldots ,y_{k}\left(t\right)\right]}^{T}$ and $\varepsilon_{1}\left(t_{i}\right),\varepsilon_{2}\left(t_{i}\right),\ldots ,\varepsilon_{k}\left(t_{i}\right)$ represent the white Gaussian noise. The accurate acquisition of the EEG signals is of great significance, and denoising of the signal is required before further processing. The multiscale principle component analysis (MSPCA) plays vital role in the denoising of a signal, which is a combination of principle component analyses and wavelet [59], and which is used for robust motor imagery brain computer interface classification [60,61]. The system of differential equations are constructed as [13]:

(2) $$\begin{array}{c} {\dot{y}}_{1}\left(t\right)=\sum_{m=1}^{k}a_{1m}y_{m}\left(t\right)+y^{T}\left(t\right)B_{1}y\left(t\right)+c_{1},\\ {\dot{y}}_{2}\left(t\right)=\sum_{m=1}^{k}a_{2m}y_{m}\left(t\right)+y^{T}\left(t\right)B_{2}y\left(t\right)+c_{2},\\ \vdots \\ {\dot{y}}_{k}\left(t\right)=\sum_{m=1}^{k}a_{km}y_{m}\left(t\right)+y^{T}\left(t\right)B_{k}y\left(t\right)+c_{k}. \end{array}$$

The (*k* × *j*) matrix of unknown coefficients *D*, matrix *Y*, and matrix $\dot{Y}$ are introduced in (3), (4), and (5), respectively:

(3) $$D=\left[\begin{array}{cccccccccc} c_{1} & a_{11} & \cdots & a_{1k} & b_{11}^{1} & \cdots & b_{kk}^{1} & 2b_{12}^{1} & \cdots & 2b_{k-1,k}^{1}\\ c_{2} & a_{21} & \cdots & a_{2k} & b_{11}^{2} & \cdots & b_{kk}^{2} & 2b_{12}^{2} & \cdots & 2b_{k-1,k}^{2}\\ \vdots & \vdots & \cdots & \vdots & \vdots & \cdots & \vdots & \vdots & \cdots & \vdots \\ c_{k} & a_{k1} & \cdots & a_{kk} & b_{11}^{k} & \cdots & b_{kk}^{k} & 2b_{12}^{k} & \cdots & 2b_{k-1,k}^{k} \end{array}\right]$$

(4) $$Y=\left[\begin{array}{cccccccccc} 1 & y_{11} & \cdots & y_{k1} & y_{11}^{2} & \cdots & y_{k1}^{2} & y_{11}y_{21} & \cdots & y_{k-1,1}y_{k,1}\\ 1 & y_{12} & \cdots & y_{k2} & y_{12}^{2} & \cdots & y_{k2}^{2} & y_{12}y_{22} & \cdots & y_{k-1,2}y_{k,2}\\ \vdots & \vdots & \cdots & \vdots & \vdots & \cdots & \vdots & \vdots & \cdots & \vdots \\ 1 & y_{1N} & \cdots & y_{kN} & y_{1N}^{2} & \cdots & y_{kN}^{2} & y_{1N}y_{2N} & \cdots & y_{k-1,N}y_{k,N} \end{array}\right]$$

(5) $$\dot{Y}=\left[\begin{array}{cccc} {\dot{y}}_{11} & {\dot{y}}_{21} & \cdots & {\dot{y}}_{k1}\\ {\dot{y}}_{12} & {\dot{y}}_{22} & \cdots & {\dot{y}}_{k2}\\ \vdots & \vdots & \cdots & \vdots \\ {\dot{y}}_{1N} & {\dot{y}}_{2N} & \cdots & {\dot{y}}_{kN} \end{array}\right]$$

Considering *k* = 3 and $c={\left[c_{1},c_{2},\ldots ,c_{k}\right]}^{T}=0$ [13]:

(6) $$\begin{array}{l} {\dot{y}}_{1}\left(t\right)=a_{11}y_{1}\left(t\right)+a_{12}y_{2}\left(t\right)+a_{13}y_{3}\left(t\right)+b_{11}^{1}y_{1}^{2}\left(t\right)+b_{22}^{1}y_{2}^{2}\left(t\right)+b_{33}^{1}y_{3}^{2}\left(t\right)\\ \;+2b_{12}^{1}y_{1}\left(t\right)y_{2}\left(t\right)+2b_{13}^{1}y_{1}\left(t\right)y_{3}\left(t\right)+2b_{23}^{1}y_{2}\left(t\right)y_{3}\left(t\right)\\ {\dot{y}}_{2}\left(t\right)=a_{21}y_{1}\left(t\right)+a_{22}y_{2}\left(t\right)+a_{23}y_{3}\left(t\right)+b_{11}^{2}y_{1}^{2}\left(t\right)+b_{22}^{2}y_{2}^{2}\left(t\right)+b_{33}^{2}y_{3}^{2}\left(t\right)\\ \;+2b_{12}^{2}y_{1}\left(t\right)y_{2}\left(t\right)+2b_{13}^{2}y_{1}\left(t\right)y_{3}\left(t\right)+2b_{23}^{2}y_{2}\left(t\right)y_{3}\left(t\right)\\ {\dot{y}}_{3}\left(t\right)=a_{31}y_{1}\left(t\right)+a_{32}y_{2}\left(t\right)+a_{33}y_{3}\left(t\right)+b_{11}^{3}y_{1}^{2}\left(t\right)+b_{22}^{3}y_{2}^{2}\left(t\right)+b_{33}^{3}y_{3}^{2}\left(t\right)\\ \;+2b_{12}^{3}y_{1}\left(t\right)y_{2}\left(t\right)+2b_{13}^{3}y_{1}\left(t\right)y_{3}\left(t\right)+2b_{23}^{3}y_{2}\left(t\right)y_{3}\left(t\right) \end{array}$$

For practical application, let the EEG signal of the subject be taken at three different points of his cerebral cortex and considered as a three-time series describing the behavior in the three-dimensional space. In standard medical procedure, measuring sensors are placed on some defined points of the cerebral cortex. For this study, we considered the magnitude of electrical impulses at points *P*3, *P*4, and *O*1, as well as *C*3, *C*4, and *T*5, designated by coordinate $y_{1}\left(t\right)$, $y_{2}\left(t\right)$, and $y_{3}\left(t\right)$. A three-dimensional system based on these time series was constructed as:

(7) $$\begin{array}{l} {\dot{y}}_{1}\left(t\right)=-20.93+1.55y_{1}\left(t\right)+6.20y_{2}\left(t\right)-7.05y_{3}\left(t\right)+0.016y_{1}^{2}\left(t\right)+0.17y_{2}^{2}\left(t\right)\\ \;-0.16y_{3}^{2}\left(t\right)-0.10y_{2}\left(t\right)y_{1}\left(t\right)+0.13y_{3}\left(t\right)y_{1}\left(t\right)-0.08y_{3}\left(t\right)y_{2}\left(t\right)\\ {\dot{y}}_{2}\left(t\right)=3.87-2.60y_{1}\left(t\right)+2.12y_{2}\left(t\right)-2.62y_{3}\left(t\right)-0.01y_{1}^{2}\left(t\right)+0.034y_{2}^{2}\left(t\right)\\ \;-0.13y_{3}^{2}\left(t\right)-0.17y_{2}\left(t\right)y_{1}\left(t\right)+0.32y_{3}\left(t\right)y_{1}\left(t\right)+0.025y_{3}\left(t\right)y_{2}\left(t\right)\\ {\dot{y}}_{3}\left(t\right)=-12.12+1.36y_{1}\left(t\right)+3.20y_{2}\left(t\right)-3.56y_{3}\left(t\right)+0.03y_{1}^{2}\left(t\right)+0.06y_{2}^{2}\left(t\right)\\ \;-0.14y_{3}^{2}\left(t\right)-0.14y_{2}\left(t\right)y_{1}\left(t\right)+0.09y_{3}\left(t\right)y_{1}\left(t\right)+0.08y_{3}\left(t\right)y_{2}\left(t\right) \end{array}$$

The system (7) simulated the impulses at *C*3, *C*4, and *T*5, while the system of the differential equation presented in (8) and (9) simulated the impulse at the *P*3, *P*4, and *O*1 points:

(8) $$\begin{array}{l} {\dot{y}}_{1}\left(t\right)=-3.11+0.19y_{1}\left(t\right)+0.72y_{2}\left(t\right)-1.19y_{3}\left(t\right)+0.022y_{1}^{2}\left(t\right)-0.04y_{2}^{2}\left(t\right)\\ \;+0.045y_{3}^{2}\left(t\right)+0.04y_{2}\left(t\right)y_{1}\left(t\right)-0.01y_{3}\left(t\right)y_{1}\left(t\right)-0.06y_{3}\left(t\right)y_{2}\left(t\right)\\ {\dot{y}}_{2}\left(t\right)=-4.58-1.69y_{1}\left(t\right)+0.39y_{2}\left(t\right)+1.37y_{3}\left(t\right)-0.03y_{1}^{2}\left(t\right)-0.09y_{2}^{2}\left(t\right)\\ \;+0.05y_{3}^{2}\left(t\right)+0.11y_{2}\left(t\right)y_{1}\left(t\right)+0.08y_{3}\left(t\right)y_{1}\left(t\right)-0.06y_{3}\left(t\right)y_{2}\left(t\right)\\ {\dot{y}}_{3}\left(t\right)=-7.88+6.91y_{1}\left(t\right)-5.69y_{2}\left(t\right)-0.71y_{3}\left(t\right)+0.23y_{1}^{2}\left(t\right)+0.025y_{2}^{2}\left(t\right)\\ \;+0y_{3}^{2}\left(t\right)-0.17y_{2}\left(t\right)y_{1}\left(t\right)-0.1y_{3}\left(t\right)y_{1}\left(t\right)+0.06y_{3}\left(t\right)y_{2}\left(t\right) \end{array}$$

(9) $$\begin{array}{l} {\dot{y}}_{1}\left(t\right)=-1.24-1.15y_{1}\left(t\right)+2.34y_{2}\left(t\right)-0.83y_{3}\left(t\right)-0.04y_{1}^{2}\left(t\right)+0y_{2}^{2}\left(t\right)\\ \;+0.02y_{3}^{2}\left(t\right)+0.235y_{1}\left(t\right)y_{1}\left(t\right)-0.015y_{3}\left(t\right)y_{1}\left(t\right)-0.12y_{3}\left(t\right)y_{2}\left(t\right)\\ {\dot{y}}_{2}\left(t\right)=3.68-3.91y_{1}\left(t\right)+1.01y_{2}\left(t\right)+2.54y_{3}\left(t\right)-0.16y_{1}^{2}\left(t\right)-0.08y_{2}^{2}\left(t\right)\\ \;+0.02y_{3}^{2}\left(t\right)+0.15y_{2}\left(t\right)y_{1}\left(t\right)+0.10y_{3}\left(t\right)y_{1}\left(t\right)-0.04y_{3}\left(t\right)y_{2}\left(t\right)\\ {\dot{y}}_{3}\left(t\right)=-5.9+5.22y_{1}\left(t\right)-6.15y_{2}\left(t\right)-0.31y_{3}\left(t\right)+0.13y_{1}^{2}\left(t\right)+0.022y_{2}^{2}\left(t\right)\\ \;+0y_{3}^{2}\left(t\right)-0.13y_{2}\left(t\right)y_{1}\left(t\right)-0.28y_{3}\left(t\right)y_{1}\left(t\right)+0.05y_{3}\left(t\right)y_{2}\left(t\right) \end{array}$$

Now, the details regarding the implementation of the proposed intelligent neuro supervised networks are presented. The proposed scheme is implemented in two steps:

- Reference dataset generation: First, the reference dataset for the INSNs is generated through determining the numerical results of the PDI models presented in (7) to (9). The state of the art Adams procedure is used to determine the numerical results of the PDI models of (7) to (9) through the ‘NDSolve’ routine of Mathematica software for finding the solution of the systems represented by the differential equations for t∈[0,5], with a step size 0.2, i.e., total 251 input (time instances), and, accordingly, a 753 output (number of measurements) with 251 discrete instances for each *y*_1_, *y*_2_, *y*_3_. The value of the parameters of the quantities of interest and initial population representing the location of sensors for electrical rhythms of the brain are taken from the reported study [13]. Further information regarding the justification of the parameter on the basis of theoretical analyses, i.e., global and local stability and population dynamics, can be seen in the reported study [13].
- Developing neuro-supervised networks: The INSNs are constructed through a neural networks structure with logistic activation function to solve the PDI models of (7) to (9). For backpropagation, two different optimization algorithms are used, i.e., Levenberg–Marquardt (LM) and Bayesian regularization (BR). In LM, the number of hidden neurons is taken as 20 for all three PDI models of (7) to (9), while in the case of BR, the number of hidden neurons for the PDI model of (7) are 50, and for remaining two PDI models of (8) and (9), the neurons are 100.

The optimizers based on LM and BR adjust the weights of the neural networks through minimizing the deviation from the reference numerical solution in the mean square error (MSE) sense. The MSE, absolute error (AE), to assess the performance of the proposed INSNs is defined as:

(10) $$MSE_{y_{1}}=mean{\left(y_{1}-{\tilde{y}}_{1}\right)}^{2};MSE_{y_{2}}=mean{\left(y_{2}-{\tilde{y}}_{2}\right)}^{2};MSE_{y_{3}}=mean{\left(y_{3}-{\tilde{y}}_{3}\right)}^{2}$$

(11) $$AE_{y_{1}}=abs\left(y_{1}-{\tilde{y}}_{1}\right);AE_{y_{2}}=abs\left(y_{2}-{\tilde{y}}_{2}\right);AE_{y_{3}}=abs\left(y_{3}-{\tilde{y}}_{3}\right)$$

The proposed INSNs may play a significant role in solving the PDI mathematical models. As PDI is a complex neurological disorder, its accurate mathematical modeling can help understand the underlying mechanisms, its progression prediction, and developing effective treatment strategies. The proposed INSNs are capable of analyzing the complex data, identifying patterns, and making predictions, and they thus may contribute in advancing the knowledge of PDI and improving patient care. Therefore, in this study, the authors proposed a neural networks-based intelligent framework for solving the PDI mathematical model. However, this framework can be extended for clinical contributions in terms of early and efficient diagnosis as well as the prediction of PDI. Moreover, the proposed INSNs can assist in optimizing PDI treatment strategies by considering various factors, such as age, symptoms, and medication history. The proposed INSNs can help predict the most effective treatment options and dosages for individual patients. This can help in enhancing personalized medicine approaches and improved patient outcomes.

The INSNs normally demand more computational requirements, especially when dealing with large datasets. Training and optimizing INSNs for PDI models may require significant computational resources and time. This may affect the practical implementation of the INSNs, particularly for researchers with limited computing resources.

## 4. Performance Analyses

The simulation results of the proposed INSNs for PDI models 1, 2, and 3 presented in (7), (8), and (9), respectively, are provided in this section by considering both the BR and LM optimization algorithms.

In order to analyze the performance of the proposed INSNs, first, a reference dataset through the Adams solver is generated for PDI models 1, 2, and 3 that are presented in (7), (8), and (9), respectively, for inputs [0, 5] with a step size of 0.025. The dataset for all three PDI models is arbitrarily segmented into training, testing, and validation, with a proportion of 80, 10, and 10, respectively. The block diagram representation of the proposed INSN layer structure is presented in part 3 in Figure 1, and is implemented in the Matlab fitting tool.

**Figure 2:**
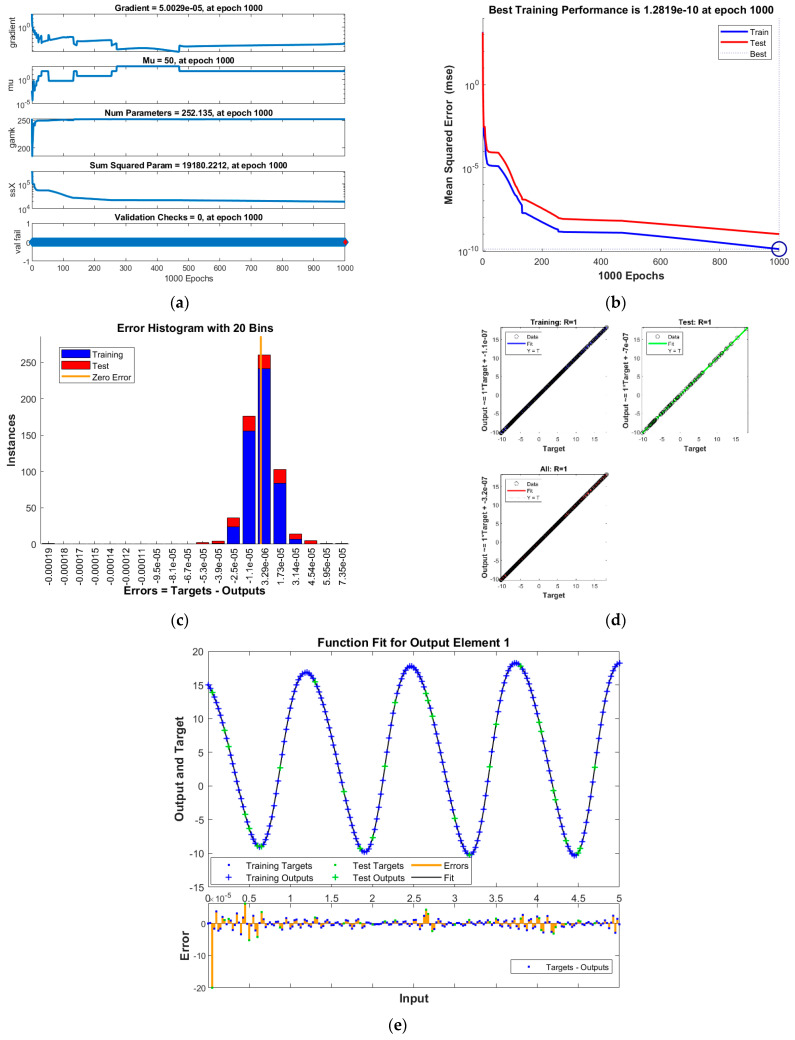
Results of INSN-BR for PDI model 1: (**a**) State transition values; (**b**) Learning curve; (**c**) Histogram; (**d**) Regression results; (**e**) Fitting results.

**Figure 3:**
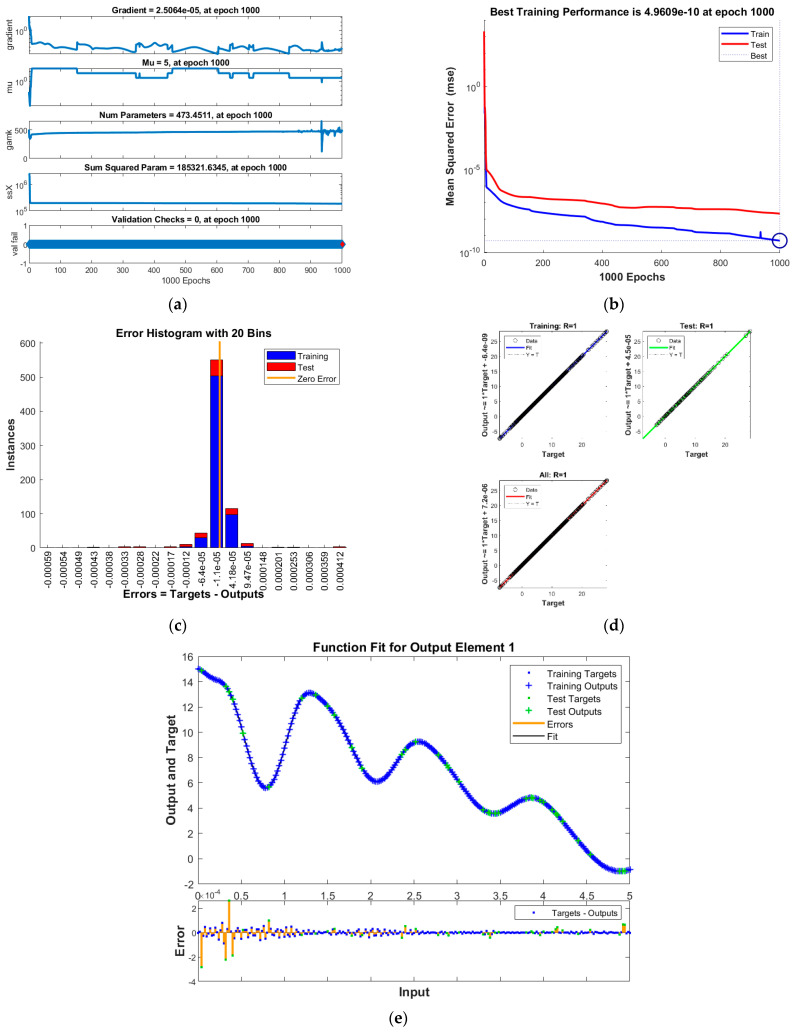
Results of INSN-BR for PDI model 2: (**a**) State transition values; (**b**) Learning curve; (**c**) Histogram; (**d**) Regression results; (**e**) Fitting results.

**Figure 4:**
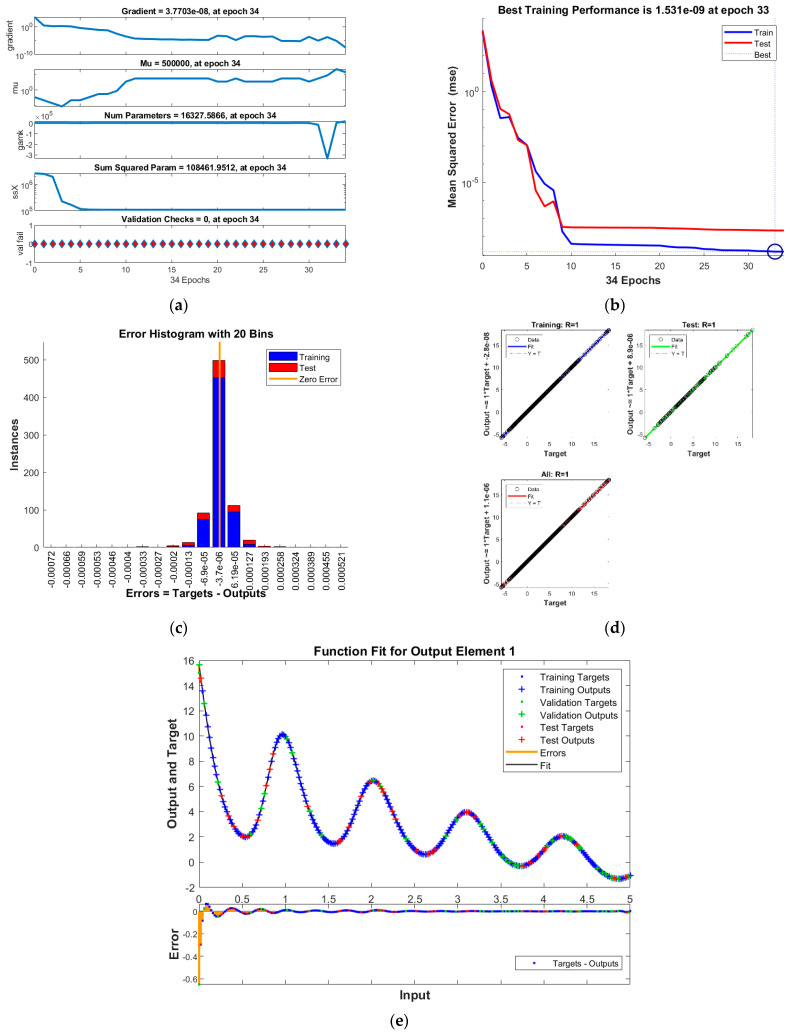
Results of INSN-BR for PDI model 3: (**a**) State transition values; (**b**) Learning curve; (**c**) Histogram; (**d**) Regression results; (**e**) Fitting results.

**Figure 5:**
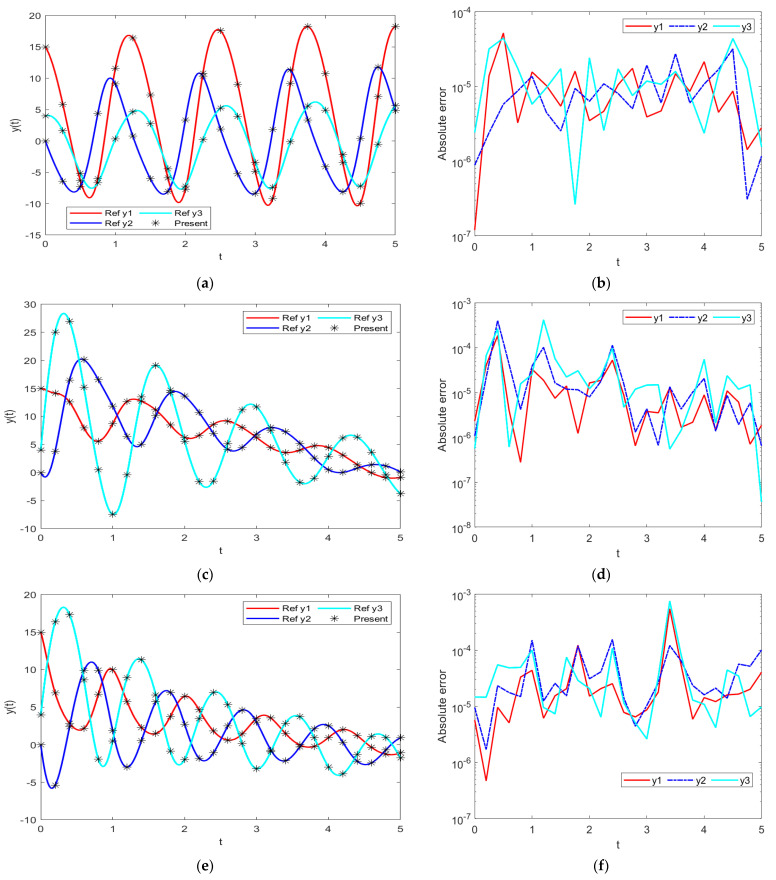
Comparative results of the proposed INSN-BR with the reference numerical solution: (**a**) PDI model 1; (**b**) PDI model 1; (**c**) PDI model 2; (**d**) PDI model 2; (**e**) PDI model 3; (**f**) PDI model 3.

**Figure 6:**
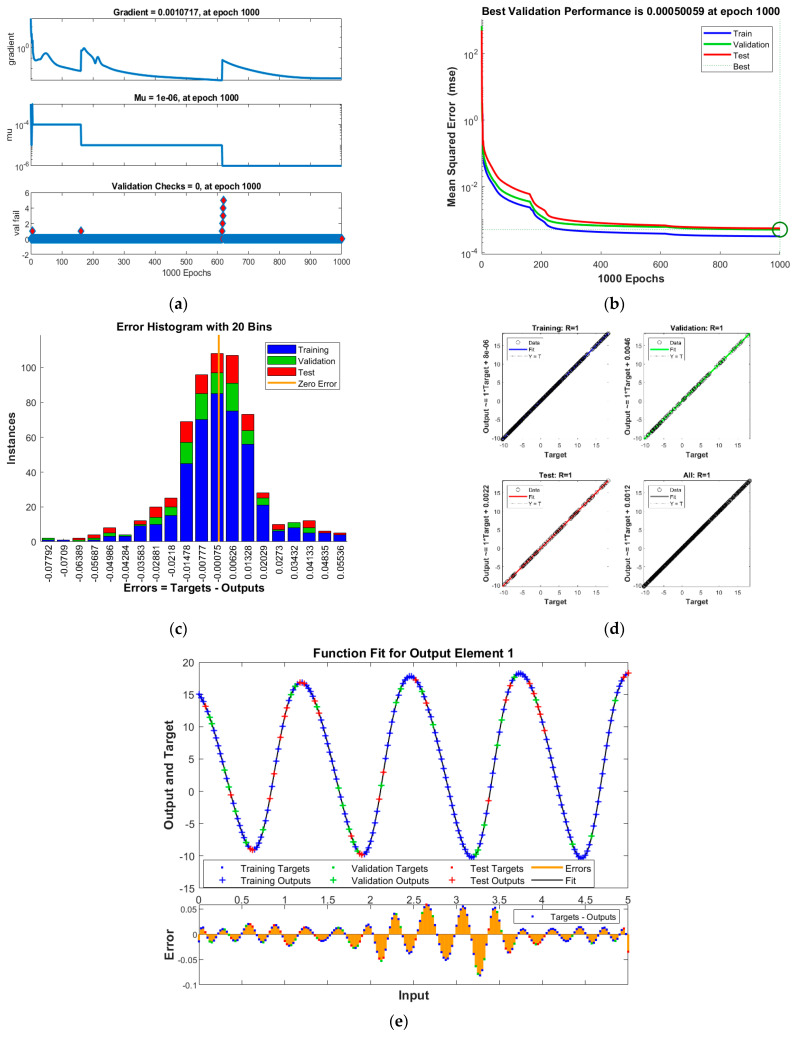
Results of INSN-LM for PDI model 1: (**a**) State transition values; (**b**) Learning curve; (**c**) Histogram; (**d**) Regression results; (**e**) Fitting results.

**Figure 7:**
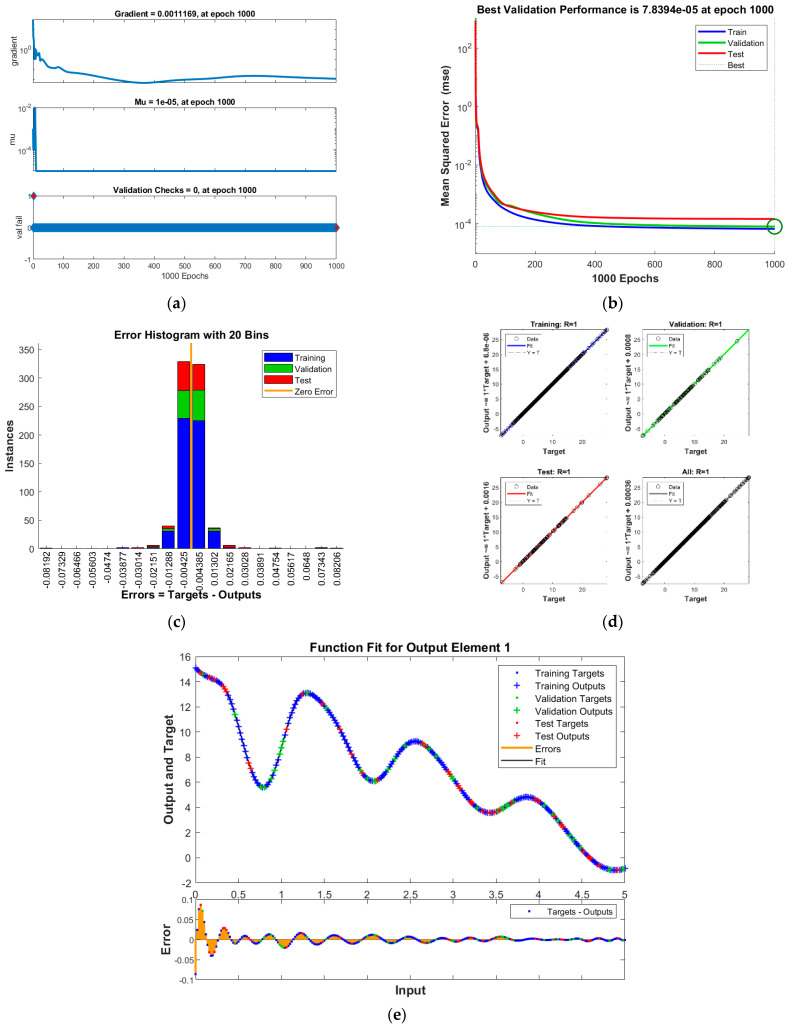
Results of INSN-LM for PDI model 2: (**a**) State transition values; (**b**) Learning curve; (**c**) Histogram; (**d**) Regression results; (**e**) Fitting results.

**Figure 8:**
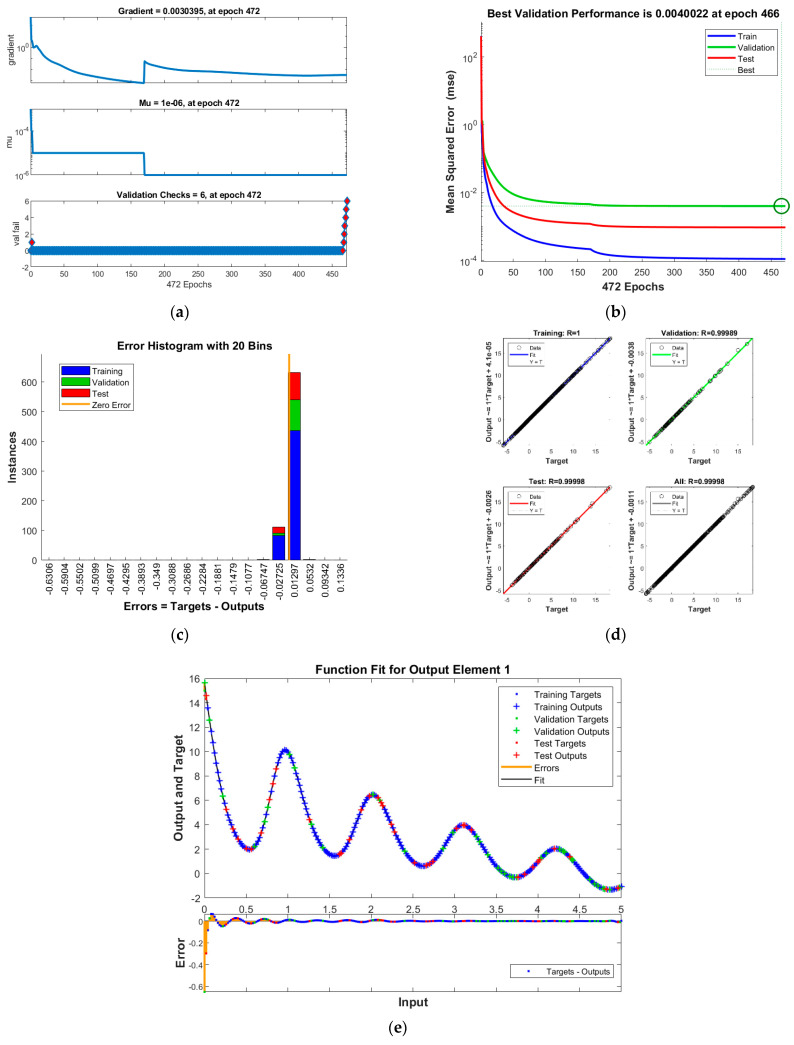
Results of INSN-LM for PDI model 3: (**a**) State transition values; (**b**) Learning curve; (**c**) Histogram; (**d**) Regression results; (**e**) Fitting results.

**Figure 9:**
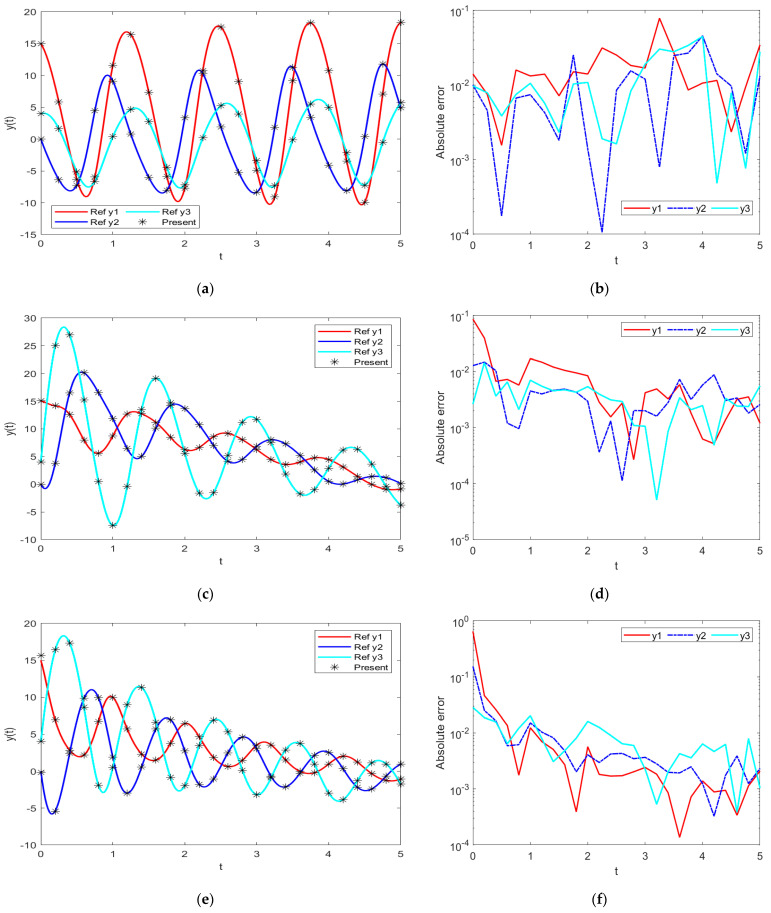
Comparative results of the proposed INSN-LM with the reference numerical solution: (**a**) PDI model 1; (**b**) PDI model 1; (**c**) PDI model 2; (**d**) PDI model 2; (**e**) PDI model 3; (**f**) PDI model 3.

The results of INSNs with BR (INSN-BR) are given in Figure 2, Figure 3, Figure 4 and Figure 5, and the results of INSNs with LM (INSN-LM) are provided in Figure 6, Figure 7, Figure 8 and Figure 9. The results of INSN-LM are provided in Figure 2, Figure 3 and Figure 4 for PDI models 1, 2, and 3, respectively. Figure 2a, Figure 3a, and Figure 4a provide the state transition values; Figure 2b, Figure 3b, and Figure 4b provide the learning curves; Figure 2c, Figure 3c, and Figure 4c present the histogram analyses; Figure 2d, Figure 3d, and Figure 4d provide the regression results; and Figure 2e, Figure 3e, and Figure 4e present the fitting results.

The best training performance of the proposed INSN-BR is 1.2819 × 10^−10^ at 1000 epochs, 4.9609 × 10^−10^ at 1000 epochs, and 1.531 × 10^−9^ at 33 epochs for PDI models 1, 2, and 3, respectively. The corresponding gradient and learning rates are [5.0029 × 10^−5^, 2.5064 × 10^−5^, 3.7703 × 10^−8^] and [50, 5 and 500,000], respectively. Further, it is observed from the histogram analyses that the bin with a reference value of zero error value has error values of around 3.29 × 10^−6^, −1.1 × 10^−5^, and −3.7 × 10^−6^ for PDI models 1, 2, and 3, respectively. Moreover, the regression results show that the value for the coefficient of determination is *R* = 1 for all three PDI models, which confirms the correctness of the proposed INSN-BR.

In order to further demonstrate the accuracy/correctness of the proposed ISNS-BR, the absolute error is calculated for all three PDI models, and the results are presented in Figure 5 along with the comparison of the proposed solutions obtained through the INSN-BR with the reference numerical solutions. The results presented in Figure 5 endorse the efficacy of the proposed INSN-BR.

The results of the proposed INSN-LM are provided in Figure 6, Figure 7 and Figure 8 for PDI models 1, 2, and 3, respectively. Figure 6a, Figure 7a, and Figure 8a provide the state transition values; Figure 6b, Figure 7b, and Figure 8b provide the learning curves; Figure 6c, Figure 7c, and Figure 8c present the histogram analyses; Figure 6d, Figure 7d, and Figure 8d provide the regression results; and Figure 6e, Figure 7e, and Figure 8e present the fitting plots.

The best validation performance of the proposed INSN-LM is 5.0059 × 10^−3^ at 1000 epochs, 7.8394 × 10^−5^ at 1000 epochs, and 4.0022 × 10^−3^ at 466 epochs for PDI models 1, 2, and 3, respectively. The corresponding gradient and the learning rates are [0.0010, 0.0011, 0.0030] and [1 × 10^−6^, 1 × 10^−5^ and 1 × 10^−6^], respectively. Further, it is observed from the histogram analyses that the bin with a reference value of zero error has error values of around −7.7 × 10^−4^, −4.25 × 10^−3^, and 1.29 × 10^−2^ for PDI models 1, 2, and 3, respectively. Moreover, the regression results show the value for coefficient of determination is *R* = 1 for all three PDI models, which confirms the correctness of the proposed INSN-LM. In order to further demonstrate the accuracy/correctness of the proposed ISNS-LM, the absolute error is calculated for all three PDI models, and the results are presented in Figure 9 along with the comparison of the proposed solutions obtained through the INSN-LM with the reference numerical solutions. The results presented in Figure 9 endorse the efficacy of the proposed INSN-LM.

**Figure 10:**
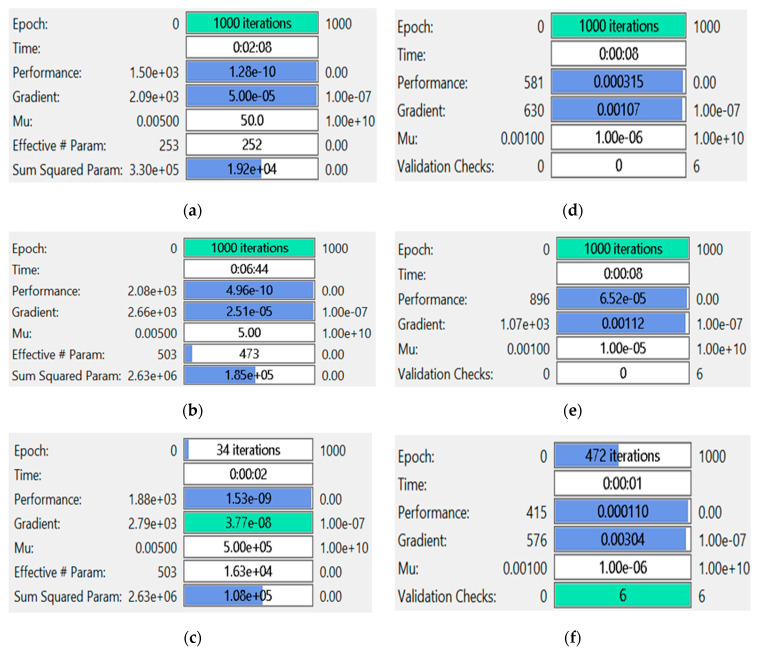
Performance comparison of INSN-BR and INSN-LM for PDI models: (**a**) INSN-BR for PDI model 1; (**b**) INSN-BR for PDI model 2; (**c**) INSN-BR for PDI model 3; (**d**) INSN-LM for PDI model 1; (**e**) INSN-LM for PDI model 2; (**f**) INSN-LM for PDI model 3.

The comparison of the INSN-BR and ISNS-LM is also conducted with respect to the MSE-based fitness values, the number of epochs, the time consumed in the computation, and the BR/LM parameters, such as gradient and learning rate, for all three PDI models, and the results are presented in Figure 10.

Figure 10a–c provides the results of INSN-BR for PDI models 1, 2, and 3, respectively, while the respective results of INSN-LM are given in Figure 10d–f, where the # means number. The INSN-BR attains the performance of 1.2819 × 10^−10^, 4.9609 × 10^−10^, and 1.531 × 10^−9^ in times of 0:02:08, 0:06:44, and 0:00:02 with 1000, 1000, and 34 epochs. The INSN-LM, meanwhile, attains the performance of 3.12 × 10^−4^, 6.54 × 10^−5^, and 1.10 × 10^−4^ in times of 0:00:08, 0:00:08, and 0:00:01 with 1000, 1000, and 472 epochs. The results clearly indicate that the INSN-BR provides more accurate results than the ISNS-LM but at the cost of bit more computation.

**Figure 11:**
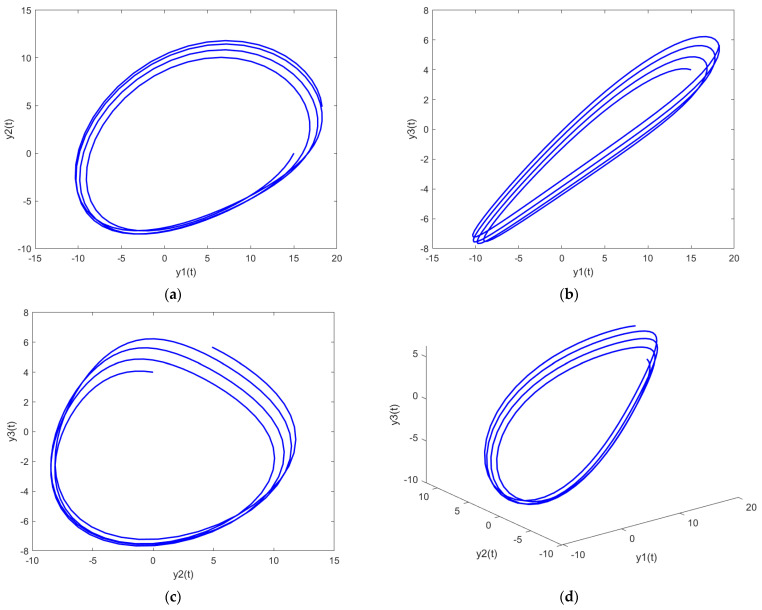
Parametric plots of PDI model 1: (**a**) y1 vs. y2; (**b**) y1 vs. y3; (**c**) y2 vs. y3; (**d**) y1 vs. y2 vs. y3.

**Figure 12:**
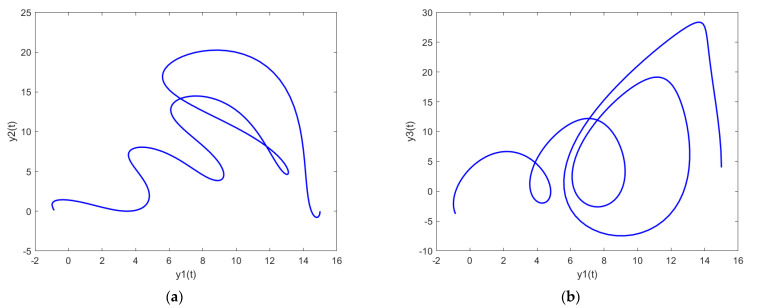

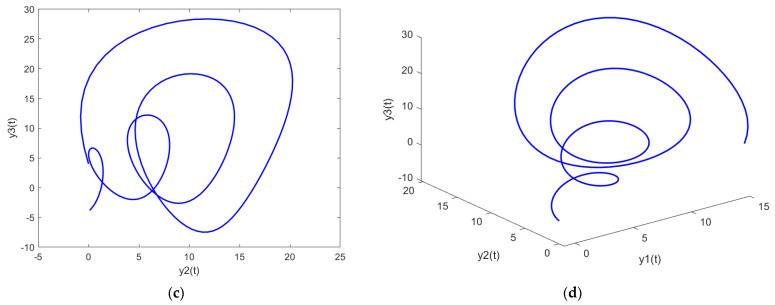
Parametric plots of PDI model 2: (**a**) y1 vs. y2; (**b**) y1 vs. y3; (**c**) y2 vs. y3; (**d**) y1 vs. y2 vs. y3.

**Figure 13:**
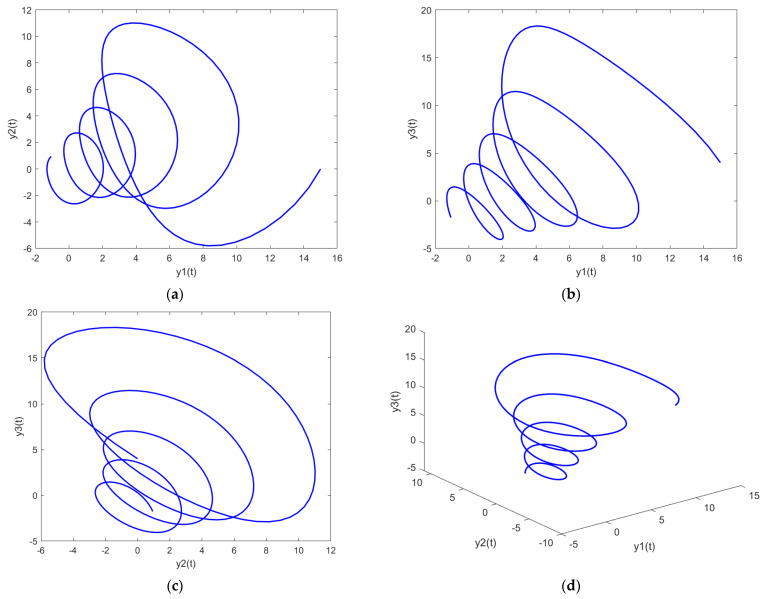
Parametric plots of PDI model 3: (**a**) y1 vs. y2; (**b**) y1 vs. y3; (**c**) y2 vs. y3; (**d**) y1 vs. y2 vs. y3.

In order to further analyze the behavior of the PDI models presented in (7) to (9), the parametric plots are also drawn and presented in Figure 11, Figure 12 and Figure 13 for PDI model 1, 2, and 3, respectively.

Figure 11a, Figure 12a and Figure 13a show the parametric plot of y1 and y2 for PDI model 1, 2, and 3, respectively. Similarly, Figure 11b, Figure 12b and Figure 13b provide the parametric plots of y1 and y2 for PDI model 1, 2, and 3, respectively, and Figure 11c, Figure 12c and Figure 13c provide the parametric plots of y2 and y3 for PDI models 1, 2, and 3. To further deepen the analyses, the 3D parametric plots are also constructed and presented in Figure 11d, Figure 12d and Figure 13d for PDI models 1, 2, and 3, respectively. The parametric plots of Figure 11, Figure 12 and Figure 13 further establish the stability of the PDI models.

## 5. Conclusions

- This study presented intelligent neuro-supervised networks, INSNs, in order to study the dynamics of Parkinson’s disease illness (PDI) through the rhythms of brain electrical activity measured at different locations on the cerebral cortex, represented with three differential classes. Two types of INSNs are constructed by neural networks multilayer architecture backpropagated with the Levenberg–Marquardt and the Bayesian regularization algorithms, i.e., INSN-LM and INSN-BR. The Adams solver is used to generate the reference data for grids of input and target samples of INSNs for different PDI models obtained by varying the sensor locations in order to measure the impact of rhythms of brain electrical activity. The dataset for all three PDI models is arbitrarily segmented into training, testing, and validation, with a proportion of 80, 10, and 10, respectively, by optimizing the fitness function based on the mean squared error criterion. The values of mean square error and absolute error endorse the accuracy and the correctness of the proposed INSN-LM and INSN-BR for all three of the PDI models. Further, the analyses by means of histogram error plots, learning curves, control parameters, and regression index all confirm the efficacy of the proposed INSNs for the PDI models, although the accuracy of INSN-RB is relatively superior to the INSN-LM, albeit at the cost of slightly more computational budget requirements.
- In future, it looks promising to incorporate the fractional gradient-based algorithms [62,63] for backpropagation in INSNs for analyzing PDI models, and to investigate early and efficient diagnosis as well as prediction of PDI through the proposed INSNs.
